# Design and evaluation of a poly-epitope based vaccine for the induction of influenza A virus cross-reactive CD8 + T cell responses

**DOI:** 10.1038/s41598-025-95479-9

**Published:** 2025-03-27

**Authors:** Sharmistha Dam, Alina Tscherne, Leoni Engels, Gerd Sutter, Albert D. M. E. Osterhaus, Guus F. Rimmelzwaan

**Affiliations:** 1 Research Center for Emerging Infections and Zoonoses, University of Veterinary Medicine, Hannover, Germany; 2 Division of Virology, Department of Veterinary Sciences, Ludwig Maximilians University Munich (LMU Munich), Oberschleißheim, Germany; 3 German Center for Infection Research (DZIF), partner site Munich, Oberschleißheim, Germany

**Keywords:** Influenza virus, Conserved epitope, CD8 + T cells, Universal vaccine, Cross-reactivity, Influenza virus, Peptide vaccines

## Abstract

The availability of influenza vaccines that can induce broadly protective immune responses is highly desirable and could also mitigate the impact of future influenza pandemics. Ideally, these vaccines also induce virus-specific CD8 + T cells, which have been identified as an independent correlate of protection. In the present study, we explored the use of an artificial immunogen that comprises of twenty highly conserved influenza virus CD8 + T cell epitopes with an HLA coverage of 99.5% of the world population. The highly attenuated viral vector Modified Vaccinia virus Ankara (MVA) was used to deliver the artificial poly-epitope sequence (rMVA-PE) and by using T cell lines raised against individual epitopes, we confirmed that the epitopes are liberated from the artificial immunogen. For efficient antigen processing and presentation, the epitopes were separated by spacer sequences. Stimulation of peripheral blood mononuclear cells of HLA-typed blood donors with rMVA-PE resulted in the activation of influenza virus-specific T cell responses. Furthermore, immunization of humanized HLA-A2.1-/HLA-DR1-transgenic H-2 class I-/class II-knockout mice (HLA-A*02:01) with rMVA-PE induced influenza virus-specific CD8 + T cell responses. Thus, rMVA-PE proved to be immunogenic both in vitro and in vivo and constitutes a promising vaccine candidate for the induction of cross-reactive CD8 + T cell responses that could afford protection against antigenically distinct influenza A viruses (IAV) of various subtypes and species, and is currently considered for further clinical testing.

**Supplementary Information:**

The online version contains supplementary material available at 10.1038/s41598-025-95479-9.

## Introduction

Influenza A (IAV) and B viruses, collectively are an important cause of respiratory disease outbreaks in humans and are notorious for their capacity to evade host immunity induced by previous infections or vaccinations^1^. Because of antigenic drift^2–4^, the composition of annual vaccines against seasonal influenza has to be updated annually. When vaccine components do not match the epidemic strains antigenically, vaccine efficacy is impaired considerably^5,6^.

In addition, the introduction of novel antigenically distinct IAV into the human population can spark pandemic outbreaks, when these viruses are efficiently transmitted from human-to-human. This has occurred four times within the last 106 years with viruses of novel subtypes originating from animal reservoirs^7^. The last influenza pandemic occurred in 2009 and was caused by genetically re-assorted swine influenza viruses of the H1N1 subtype^8^. Furthermore, highly pathogenic avian influenza viruses (HPAIV) have been shown to be able to infect humans, not seldomly with fatal outcome^1^. The continuous circulation of HPAIVs of the H5 subtype and their capacity to infect mammalian species is of concern and these viruses may be considered to be at the origin of the next pandemic.

To mitigate the impact of a future influenza pandemic, it is important to develop vaccines that, like seasonal influenza vaccines, antigenically match the emerging pandemic strain. However, this may take too long to mitigate the emerging pandemic significantly, as was the case in 2009, when matching pandemic vaccines became available around or even after the peak of the pandemic^9^. Therefore, for both epidemic and pandemic preparedness, the availability of vaccines that can induce broadly protective immunity against a variety of IAVs is highly desirable and has been a research focus during the last two decades^10–13^.

Currently used conventional vaccination strategies aim at the induction of virus neutralizing (VN) antibodies predominantly directed against the globular head domain of the viral hemagglutinin (HA), which is known to be highly variable^4,6,14,15^. As a strategy to achieve broadly protective immunity, the induction of antibodies to the more conserved stalk region of the HA molecule has attracted considerable interest and has shown promise^16–21^.

Another independent correlate of broadly protective immunity against influenza is the presence of CD8 + cytotoxic T cells directed against conserved viral epitopes, predominantly located in the nuclear protein (NP), matrix 1 protein (M1) and polymerases (PB1, PB2, PA)^22–25^. The main function of these T cells is to recognize and eliminate virus-infected cells, thus contributing to clearance of the virus and elimination of the infection. This has been demonstrated in various animal models but also in humans that were infected with the pandemic strain of 2009 and with HPAIV of the H7N9 subtype^23,26–29^. In these studies, the frequency of pre-existing cross-reactive memory T cells induced by previous infections with seasonal IAV inversely correlated with disease severity.

Currently used inactivated influenza vaccines induce CD8 + T cell responses inefficiently at best, because they fail to mediate endogenous antigen processing and presentation, which is required for efficient induction of virus-specific CD8 + T cell responses^30,31^. Furthermore, the currently used seasonal influenza subunit vaccines only contain minor amounts of NP and M1, if at all. Thus, for the systemic induction of CD8 + T cell responses other vaccination strategies are required, like the use of live vectors, live vaccines or vaccines with specialized adjuvant systems. For example, the Modified Vaccinia virus Ankara (MVA) has been found a suitable vector system for the induction of CD8 + T cell responses to the influenza virus NP and M1 protein in animal models and in humans^32^.

In the present study, we designed and evaluated an artificial poly-epitope sequence comprising of potentially immunogenic conserved IAV CD8 + T cell epitopes. To this end, 20 epitopes were selected from the Immune Epitope Database (IEDB), based on their high degree of conservation in human, swine and avian influenza A viruses on the one hand, and high HLA coverage on the other. In addition, the poly-epitope sequence was fused to ubiquitin and each epitope sequence was separated by a spacer sequence to ensure optimal proteolytic degradation by the proteasome as was described previously for HIV poly-epitope and SARS-CoV-2 immunogens^33,34^. For the expression of the artificial poly-epitope gene, we used the highly attenuated viral vector MVA, because of its excellent safety profile and its capacity to induce both humoral and cell mediated immune responses^35,36^. Our recombinant MVA expressing the artificial poly-epitope sequence (rMVA-PE) was antigenic and immunogenic *in* v*itro* and induced influenza virus specific CD8 + T cell responses in humanized HLA-A2.1-/HLA-DR1-transgenic H-2 class I-/class II-knockout mice. It was concluded that the rMVA-PE holds promise as an adjunct to conventional seasonal influenza vaccines and may be also considered in pandemic preparedness plans to mitigate clinical outcome of influenza virus infections until tailor made vaccines that match the pandemic strain, become available.

## Materials and methods

### Design of artificial IAV poly-epitope immunogens

For the design of an artificial poly-epitope based vaccine, 20 IAV CD8 + T cell epitopes were selected. To this end, all 972 8-11mer influenza virus CD8 + T cell epitopes were retrieved from the IEDB; www.iedb.org, accessed September 2020). From these, the top 20 epitopes were selected based on the following criteria: the highest degree of conservation in influenza viruses of human, avian or swine origin and confirmation of the antigenicity of the epitopes by showing that peptides corresponding to the epitopes were able to induce interferon-gamma (IFN-γ) secretion by virus-specific CD8 + T cells in IFN-γ ELIspot assays or by intracellular cytokine staining. In addition, HLA coverage of the artificial poly-epitope gene was taken into account and the HLA restriction elements of the respective top twenty epitopes was arbitrarily set at 99.5% of the human world population.

**Fig. 1:**
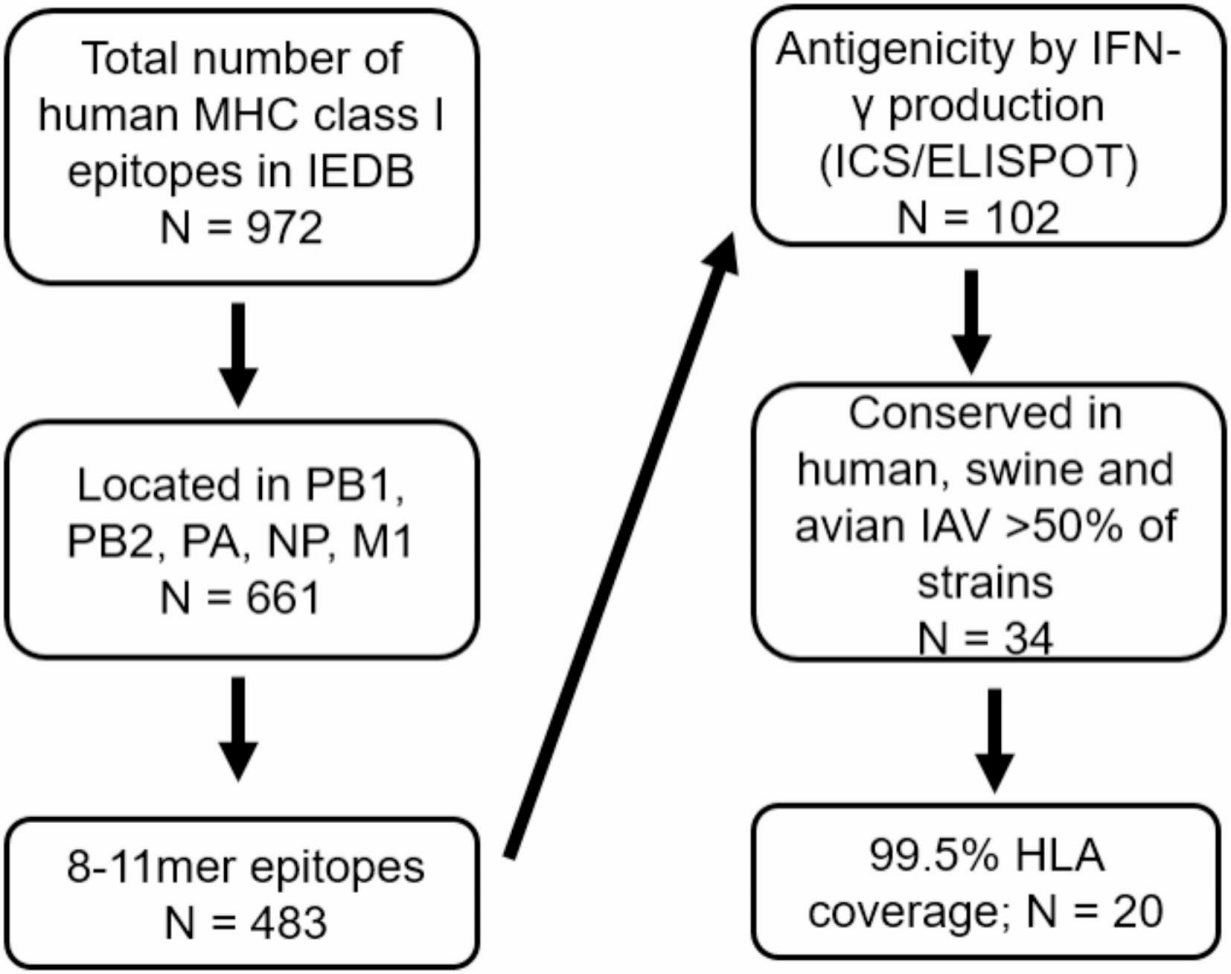
Stepwise selection of human MHC class I epitopes from the Immune Epitope Database (IEDB) for inclusion in the poly-epitope construct. 972 epitopes were retrieved from the IEDB using search terms linear peptide, IAV (ID: 11320), assay for T cell ligand, MHC Ligand, MHC restriction: class I, Host: human. IEDB accessed on: September 2020. Subsequently, 661 epitopes were located in the internal proteins PB1, PB2, PA, NP, and M1. Among these, 483 epitopes were 8–11 amino acids in length. Only epitopes were considered that induced IFN-γ responses in PBMC and that were highly conserved in human, swine and avian IAV (conserved in > 50% of all strains in the NCBI Influenza Virus database, see Table 1). From these epitopes, 20 were selected accounting for 99.5% HLA coverage of the world population.

To ensure optimal degradation of the poly-epitope protein, the encoding sequence was fused to that of ubiquitin to facilitate docking of the proteins to the proteasome^37–40^. To facilitate efficient liberation of the epitopes by proteasomal degradation and to prevent formation of neo-epitopes, the epitopes were separated by the spacer sequence AAY^41^. At the C-terminus, a HA-tag (YPYDVPDYA) was added to facilitate in vitro detection of the target antigen. Upstream and downstream of the coding sequence, a Kozak sequence and stop codon were added, respectively. For cloning purposes, BamHI and PmeI restriction sites were introduced at the 5′ and 3′ end, respectively. For expression by the poxviral vector MVA the coding sequence was optimized by interrupting G/C-runs (CCCC or GGGG) and poxvirus early transcription termination signals (TTTTTNT) were modified at the genomic level without affecting the amino acid sequence^42^. A schematic representation of the construct design is shown in Fig. 1. The sequence was synthesised (GenScript, Rijswijk, The Netherlands) and cloned into the MVA shuttle vector plasmid pIIIH5redK1L^43^under transcriptional control of the synthetic vaccinia virus (VACV) early/late PmH5 promoter^42^.

**Table 1:** Selected epitopes included in the artificial poly-epitope Immunogen. Indicated are their positions within the poly-epitope sequence, their HLA restriction, and conservation amongst human, avian and swine IAV.

Position in poly-epitope	CD8 + Epitope	Sequence and Reference	HLA restriction	Conservation in influenza viruses		
				Human	Avian	Swine
1	NP _265−274_	ILRGSVAHK^77,78^	A*03:01	98%	94%	98%
2	PA _282−290_	FLLMDALKL^79^	A*02:01	94%	97%	98%
3	NP _383−391_	SRYWAIRTR^80,81^	B*27:05,B*27:03	56%	92%	72%
4	NP _44−52_	CTELKLSDY^78,80^	A*01:01,A*30:03,A*15:17	55%	65%	72%
5	M1 _58−66_	GILGFVFTL^79,82^	A*02:01	97%	85%	97%
6	M1 _13−21_	SIIPSGPLK^83^	A*11:01	55%	54%	65%
7	M1 _128−135_	ASCMGLIY^81,82^	B*35:01	97%	94%	97%
8	PA _46−54_	FMYSDFHFI^79,82^	A*02:01,A*68:02	99%	99%	99%
9	PA _4−12_	FVRQCFNPM^84,85^	B*15:01	98%	98%	98%
10	PB1 _591−599_	VSDGGPNLY^80,81^	A*01:01	98%	96%	92%
11	PB1 _166−174_	FLKDVMESM^86^	A*02:01,B*07:02,B*15:01	94%	79%	85%
12	NP _11−19_	RGINDRNFW^86,87^	B*58:01	99%	92%	99%
13	PB1 _407−415_	MMMGMFNML^22^	A*02:01	99%	99%	99%
14	PB1 _30−38_	YSHGTGTGY^88^	A*26:01,B*15:01	99%	99%	99%
15	NP _140−148_	HSNLNDATY^88^	A*01:01,A*26:01	83%	97%	97%
16	NP _174−184_	RRSGAAGAAVK^77,80^	B*27:01	97%	93%	96%
17	M1 _3−11_	LLTEVETYV^89^	A*02:01	96%	94%	98%
18	M1 _179−187_	MVLASTTAK^90^	A*11:01	97%	95%	79%
19	PB1 _413−421_	NMLSTVLGV^88,91^	A*02:01	99%	99%	99%
20	NP _380−388_	ELRSRYWAI^77,82^	B*08:01,A*02:01	56%	94%	72%

### Cells and viruses

Primary chicken embryo fibroblasts (CEFs) were prepared from 10 to 11 days old specific pathogen free (SPF) embryonated chicken eggs^44^ (VALO BioMedia, Osterholz-Scharmbeck, Germany), which were passaged once before use, and cultured in Eagle’s minimum essential medium (EMEM; Sigma-Aldrich, St. Louis, MO, USA) supplemented with 10% heat-inactivated fetal bovine serum (FBS; Gibco, Waltham, MA, USA), 1% penicillin-streptomycin (P/S; Sigma-Aldrich, St. Louis, MO, USA), and 1% non-essential amino acids (NEAAs; Sigma-Aldrich, St. Louis, MO, USA) at 37 °C and 5% CO_2_.

A549 cells (ATCC CCL-185) were maintained in Ham’s F-12 K Nutrient Mixture (Gibco, Waltham, MA, USA) supplemented with 10% FBS, 1% P/S (Gibco, Waltham, MA, USA), and 1% GlutaMAX (Gibco, Waltham, MA, USA). Transgenic A549 cells stably expressing either HLA-A*02:01, HLA-A*01:01 or HLA-B*15:01 genes were generated previously^45^ and maintained in Ham’s F-12 K Nutrient Mixture (Gibco, Waltham, MA, USA) supplemented with 10% FBS, 1% P/S (Gibco, Waltham, MA, USA), 1% GlutaMAX (Gibco, Waltham, MA, USA) and 1 µg/ml puramycin (Gibco, Waltham, MA, USA).

Wild-type MVA (wt-MVA; clonal isolate F6)^46,47^and recombinant MVA expressing the green fluorescent protein (GFP) under transcriptional control of the VACV late promoter P11 (rMVA-GFP)^48,49^were obtained from the repository of the Institute for Infectious Diseases and Zoonoses, LMU Munich, Germany and propagated in primary CEFs. Viral titers of wt-MVA, rMVA-GFP and all recombinant MVAs (rMVAs) were determined in CEFs following established protocols as published elsewhere^43^.

Influenza virus A/Ned/427/98 (Ned98) (H3N2) was propagated and titrated in Madin-Darby canine kidney (MDCK) cells. MDCK cells were cultured in Dulbecco’s modified Eagle’s medium (Gibco, Waltham, MA, USA) supplemented with 10% FBS, 1% P/S, 1% NEAAs (Gibco, Waltham, MA, USA), and 1% GlutaMAX. MDCK cells were kept at 37⁰C and 5% CO_2_.

All cells and viruses were tested negative for mycoplasma.

### Generation of Recombinant MVA

CEFs were seeded at a density of 5 × 10^5^ cells per well into a flat-bottom 6-well plate (Corning, Corning, NY, USA). The next day, cells were infected with rMVA-GFP at a multiplicity of infection (moi) of 0.05 and incubated for 1 h at 37 °C. The virus inoculum was aspirated and replaced with infection medium consisting of EMEM supplemented with 1% P/S, 1% NEAAs, and 2% heat-inactivated FBS. 1 µg of pIIIH5redK1L-poly-epitope (containing the red fluorescent marker gene mCherry) or pIIIH5redK1L-M1 was mixed with 4 µL of X-tremeGENE HP DNA transfection reagent (Roche, Basel, Switzerland) in 100 µL of serum-free EMEM and incubated for 15 min at RT and the mixture was added dropwise to the CEFs, which were then further incubated for two days at 37 °C. Red fluorescent (mCherry-positive) foci were picked and plaque-purified until green fluorescent (GFP-positive) foci were completely lost. Subsequently, non-fluorescent (mCherry-negative) foci were picked and plaque-purified until the red fluorescent (mCherry-positive) foci were no longer detectable. Recombinant MVA carrying the poly-epitope sequence with AAY spacer sequences (rMVA-PE) was propagated in CEFs and stored at −20 °C.

Vaccine preparations were generated by amplification of the primary stock viruses in CEFs and concentration by ultracentrifugation (30,000× g, 2 h, 4 °C) through 36% (w/v) sucrose cushions. Virus pellets were resuspended in Tris-buffer (120 mM NaCl, 10 mM Tris–HCl, pH 7.4), aliquoted, and stored at − 80 °C until further use. Viral titres were determined by immuno-plaque assay using a rabbit polyclonal anti-VACV (Lister strain) antibody (OriGene, Rockville, MD, USA; 1:2000), as described previously^43^.

To confirm proper insertion of the poly-epitope sequence into deletion site III (del III) of the MVA genome, viral genomic DNA was extracted from all rMVA stock preparations using the NucleoSpin Blood QuickPure kit (Macherey-Nagel, Düren, Germany) and analysed by PCR using GoTaq Master Mix (Promega, Fitchburg, WI, USA) along with the del III–specific primers MVA-III-5′ (5′-GAATGCACATACATAAGTACCGGCATCTCTAGCAGT-3′) and MVA-III-3′ (5′-CACCAGCGTCTACAT GACGAGCTTCCGAGTTCC-3′)^43^. Viral genomic DNA of wt-MVA was used as empty vector control. To confirm identity of the transgene, the PCR product was gel-purified and submitted to sequencing (Microsynth Seqlab, Göttingen, Germany).

### Immunostaining of MVA-infected cells

CEFs grown in 6-well tissue plates at 90–95% confluency were infected with rMVAs or wt-MVA (moi 0.5), incubated for 48 h and subsequently fixed with ice-cold methanol: acetone (1:1) for 5 min. Plates were blocked with phosphate-buffered saline (PBS) (+ 3% FBS) for 1 h at RT or 4 °C o/n. Plates were washed 3x with PBS. Primary anti-VACV A27L (OriGene, Rockville, MD, USA; 1:2000) or anti-HA-tag antibodies (Thermo Fisher Scientific, Planegg, Germany; 1:1000) were diluted in PBS (+ 3% FBS), added to the wells and incubated for 1 h at RT. Subsequently, plates were washed 3x with PBS. Secondary goat anti-rabbit HRP-labeled antibody (Invitrogen, Waltham, MA, USA; 1:5000) or goat anti-mouse HRP (Invitrogen, Waltham, MA, USA; 1:5000) were diluted in PBS (+ 3% FBS), added to the wells and incubated for 1 h at RT. Afterwards, plates were washed 3x with PBS and TrueBlue™ Peroxidase Substrate (SeraCare, Milford, MA, USA) was added to each well until color change could be observed.

### Isolation of peripheral blood mononuclear cells (PBMCs)

Heparinized blood was obtained from healthy blood donors between April and August 2019 at the Blood bank of the Hannover Medical School.

PBMCs were isolated by density gradient centrifugation (Lymphoprep; Stem cell, Vancouver, BC, Canada) according to the manufacturer’s instructions and frozen in 90% FBS (Gibco, Waltham, MA, USA), 10% dimethyl sulfoxide (DMSO, Carl Roth, Karlsruhe, Germany), and cryopreserved in liquid nitrogen or at −150 °C until use. For use in subsequent experiments, PBMCs were thawed in complete RPMI 1640 medium, supplemented with P/S, Glutamax, vitamins, non-essential amino acids, sodium pyruvate (all at 1% v/v, Thermo Fisher Scientific, Planegg, Germany), 10% (v/v) heat-inactivated FBS (Gibco, Waltham, MA, USA) (R10F) and 50 µg/ml of DNAse (Sigma Aldrich, St. Louis, MO, USA).

The list of blood donors, along with their dates of birth and HLA haplotypes, is presented in Table 2.

**Table 2:** Study subjects used and epitopes examined in the present study.

PBMC donor # (HLA haplotype)	Date of birth	CD8 + epitope	Epitope sequence	HLA restriction
2534 (A*01, A*29; B*27, B*44) 2731 (A*01, A*68; B*40, B*51)	17.04.1956 19.10.1957	NP_44−52_ PB1_591−599_	CTELKLSDY VSDGGPNLY	A*01:01
2804 (A*02, A*03; B*07, B*08) 2849 (A*02, A*24; B*07, B*60)	05.08.1997 26.10.1997	M1_58−66_	GILGFVFTL	A*02:01
4843 (A*02, A*31; B*7, B*15)	02.10.1996	PB1_30−38_	YSHGTGTGY	B*15:01

### Peptides

Peptides with > 90% purity were obtained from Proimmune (Oxford, UK). Peptides were dissolved in DMSO or water depending on the hydrophobic or hydrophilic nature of the peptide. Peptides were further diluted in water to a concentration of 100 mM per peptide, aliquoted, and stored at −80⁰C until use. The final concentration of peptides was 10 µM.

### Expansion of specific CD8 + T cells by stimulation with peptides, IAV or rMVA-PE

For the expansion of peptide-specific CD8 + T cells, PBMCs from healthy HLA matched blood donors were selected for their strong response to the respective peptides, and were stimulated with 10 µM corresponding peptide (Proimmune, Oxford, UK) at 37 °C and 5% CO_2_. PBMCs of blood donors positive for HLA-A*01:01 were stimulated with peptides NP_44−52_ (CTELKLSDY) and PB1_591−599_ (VSDGGPNLY), those positive for HLA-A*02:01 with peptides M1_58−66_ (GILGFVFTL) and those positive for HLA-B*15:01 with peptide PB1_30−38_ (YSHGTGTGY). The selection of epitopes was based on the availability of PBMC donors and the presence of matching antigen-presenting cells in our laboratory.

After 3 days, IL-2 (50U/ml, PROLEUKIN^®^S, Novartis, Basel, Switzerland) was added. After 10–11 days of expansion, the peptide-specific CD8 + T cells were sorted by Magnetic-activated cell sorting (MACS) using CD8 + T cell beads (Miltyeni Biotec, Bergisch Gladbach, Germany). In addition to the use of peptides, PBMCs were stimulated with IAV Ned98 (H3N2) at a moi of 1 to expand virus-specific CD8 + T cells as described above. CD8 + T cells expanded after stimulation with peptide or IAV were used to demonstrate in vitro antigenicity of the rMVA-PE construct as described below. To demonstrate the in vitro immunogenicity of rMVA-PE, PBMCs were stimulated with either wt-MVA or rMVA-PE or IAV Ned98 at a moi of 1 or 3, and the expansion of IAV-specific T cells was demonstrated by IFN-γ ELISpot assay after re-stimulation with peptide or IAV.

### IFN-γ elispot assay

For the detection of peptide- or virus-specific T cells an IFN-γ ELISpot assay was conducted (Human IFN-ELISpot^PLUS^ kit, Mabtech, Nacka Strand, Sweden) according to the manufacturer’s instructions. To demonstrate the antigenicity of the rMVA-PE construct, MACS isolated CD8 + T cells obtained after stimulation of PBMCs with peptide or IAV Ned98 (H3N2), were re-stimulated for 20 h at 37 °C and 5% CO_2_ with HLA-transgenic A549 cells infected with rMVA-PE or wt-MVA. Mock treated or peptide-loaded A549 cells served as negative and positive controls, respectively. In addition to HLA-transgenic A549 cells, also autologous monocytes were tested. To this end, monocytes were isolated from PBMCs by using CD14 microbeads (Miltyeni Biotec, Bergisch Gladbach, Germany) and were treated as described for the A549 cells. To demonstrate in vitro immunogenicity, PBMCs were stimulated with either rMVA-PE or wt-MVA and for comparison, with IAV Ned98. The expanded and MACS-isolated CD8 + T cells were subsequently re-stimulated with HLA matched transgenic A549 cells loaded with the respective peptides and IFN-γ production was assessed by IFN-γ ELISpot assay. After development, plates were scanned, and spots were counted using the ImmunoSpot S6 Ultimate Reader and ImmunoSpot Software (Version 7.0.20.0, Immunospot, CTL). Data are presented as mean spot forming-unit (SFU) per 10^6^ PBMCs after subtraction of the values obtained with mock-treated stimulator cells.

### Mouse immunizations and splenocyte isolation

Specific-pathogen free humanized HLA-A2.1-/HLA-DR1-transgenic H-2 class I-/class II-knockout mice were obtained from Institute Pasteur/Charles River Laboratories (France)^50^ and bred in the BSL-2 animal facility of the Institute for Infectious Diseases and Zoonoses, LMU Munich, Germany. Groups of mice (*n* = 6) were anesthetized with 2% isoflurane and immunized twice (21-day interval) intramuscularly (i.m.) with 10^7^ PFU of wt-MVA, rMVA-PE or vaccination buffer in a 25 µl volume into the quadriceps muscle of the left hind leg. We chose the i.m. application, as it is the standard application route for MVA-based vaccines in humans. In addition, an immunization dose of 10^7^PFU was well tolerated and highly immunogenic in our previous studies^51,52^ using recombinant MVA vaccines and therefore also used for this study. At the end of the experiment on day 35, mice were anesthetized with 5% isoflurane and euthanized by cervical dislocation. Spleens were harvested immediately after euthanasia, processed into single-cell suspensions using the gentleMACS Octo Dissociator (Miltenyi Biotec, Bergisch Gladbach, Germany) and successively passed through 100 μm and 70 μm cell strainers (Miltenyi Biotec, Bergisch Gladbach, Germany). Erythrocytes were lysed with ACK lysing buffer (Gibco, Waltham, MA, USA) for 1.5 min at RT, followed by washing with cold PBS containing 2% FBS. Splenocytes were eventually resuspended in RPMI 1640 (Gibco, Waltham, MA, USA) supplemented with 10% FBS, 1% P/S (R10F) and kept on ice until further use.

### Mouse IFN-γ elispot

Splenocytes (2.5 × 10^5^ cells per well) were incubated in triplicate with M1_58−66_ peptide (GILGFVFTL) derived from the IAV M1 protein or the VACV A6L_6−14 _peptide (VLYDEFVTI)^53^ (2 µg/mL) in pre-coated 96-well plates (Mouse IFN-ELISpot^PLUS^ kit, Mabtech, Nacka Strand, Sweden) for 28 h at 37⁰C. Cells incubated with Phorbol 12-myrstate 13-acetate (PMA)/ionomycin (both Cayman Chemical Company, Ann Arbor, MI, USA) or DMSO were used as positive and negative controls, respectively. Plates were developed according to the manufacturer’s instructions. Developed plates were scanned using an ImmunoSpot S6 Ultimate M2 reader and spots were counted using the ImmunoSpot software version 7.0.9.5 (Version 7.0.20.0, Immunospot, CTL). Data are presented as mean spot forming-unit (SFU) per 10^6^ splenocytes after subtraction of the values obtained with the mock-treated splenocytes (1% DMSO) which served as negative control.

### Intracellular cytokine staining (ICS) and flow cytometry

Splenocytes were transferred into round-bottom 96-well plates (Sarstedt, Nuembrecht, Germany) (10^6^/well) and incubated with peptides M1_58−66_ or A6L_6−14_ (10 µg/ml) (for 6 h) or IAV HK68 (moi 1) (for 12 h) at 37⁰C, with brefeldin A (10 µg/mL; Sigma-Aldrich, St. Louis, MO, USA) added for the last 4 h. Cells incubated with PMA/ionomycin or DMSO were used as positive and negative controls, respectively. Subsequently, cells were washed with PBS/2% FBS, and then stained with LIVE/DEAD fixable near-IR dead cell stain (Invitrogen, Waltham, MA, USA) for 20 min at RT, washed twice with PBS and then incubated for 20 min with anti-CD16/CD32 (clone 93, Invitrogen, Waltham, MA, USA; 1:500) to block non-specific binding to Fc receptors and surface-stained with anti-CD3e FITC (clone 145-2C11, Invitrogen, Waltham, MA, USA), anti-CD4 PE (clone RM4-5, Invitrogen, Waltham, MA, USA), and anti-CD8a PerCP-Cy5.5 (clone 53 − 6.7, eBioscience, San Diego, CA, USA) for 20 min. All incubations were performed at RT in the dark. Subsequently, cells were fixed and permeabilised using BD Cytofix/Cytoperm solution (BD Biosciences, San Jose, CA, USA), and then stained with anti-IFN-γ APC (clone XMG1.2, Invitrogen, Waltham, MA, USA). All fluorochrome-labelled antibodies were used at a dilution of 1:200 in BD Brilliant Stain buffer (BD Biosciences, San Jose, CA, USA). Samples were acquired on a BD LSRFortessa X-20 flow cytometer (BD Biosciences, San Jose, CA, USA). Data analysis was performed using FlowJo software version 10.8.2 (BD Biosciences, San Jose, CA, USA). Values obtained with unstimulated cells were subtracted from those obtained after stimulation with peptide or virus.

### Ethics statement

All animal experiments were performed in the BSL-2 animal facility of the Institute for Infectious Diseases and Zoonoses, LMU Munich, Germany and handled in compliance with the European and national regulations for animal experimentation (European Directive 2010/63/EU; Animal Welfare Acts in Germany). Approval was obtained from regional animal ethics authorities (Regierung von Oberbayern, Munich, Germany). The study is reported in accordance with ARRIVE guidelines.

The collection of blood from healthy blood donors at the Blood bank of the Hannover Medical School (Medizinische Hochschule Hannover, MHH) was approved by the local MHH ethical committee (Permit number 3393–2016) and the subjects gave written informed consent. The work described here has been carried out in accordance the declaration of Helsinki.

### Statistical analysis

For statistical analysis, GraphPad Prism software (version 9.0.0, GraphPad Software Inc., Boston, Massachusetts, USA) was used. For all statistical tests, either Two-way ANOVA and Tukey’s multiple comparison test or the non-parametric Mann-Whitney U test for unpaired samples was used to compare experimental groups. A p-value < 0.05 was considered statistically significant.

## Results

### Selection of the epitopes

From the IEDB (accessed September 2020), a total of 972 unique influenza virus CD8 + T cell epitopes were retrieved. Since the majority of IAV-specific CD8 + T cells target internal structural proteins and polymerases, only epitopes from NP, M1, PB1, PB2, and PA were considered, narrowing the selection to 661 epitopes.

Because the preferred length of CTL epitopes presented by HLA-class I molecules ranges from 8 to 11 amino acids, only 8–11 mer epitopes were considered (*N* = 483). The antigenicity of 102 of these epitopes was demonstrated by IFN-γ ELIspot assay or intracellular cytokine staining as shown in IEDB. Thirty-four of these epitopes were found to be highly conserved using a conservation cut-off of 50% across IAV of human, swine and avian origin. Ultimately, the top 20 epitopes were selected to achieve an HLA-coverage of 99.5% of the human population. The stepwise selection process is summarized in Fig. 1. The list of the selected 20 epitopes, their HLA restriction and conservation in IAV of various species in shown in Table 1.

### Characterization of rMVA-PE constructs

**Fig. 2:**
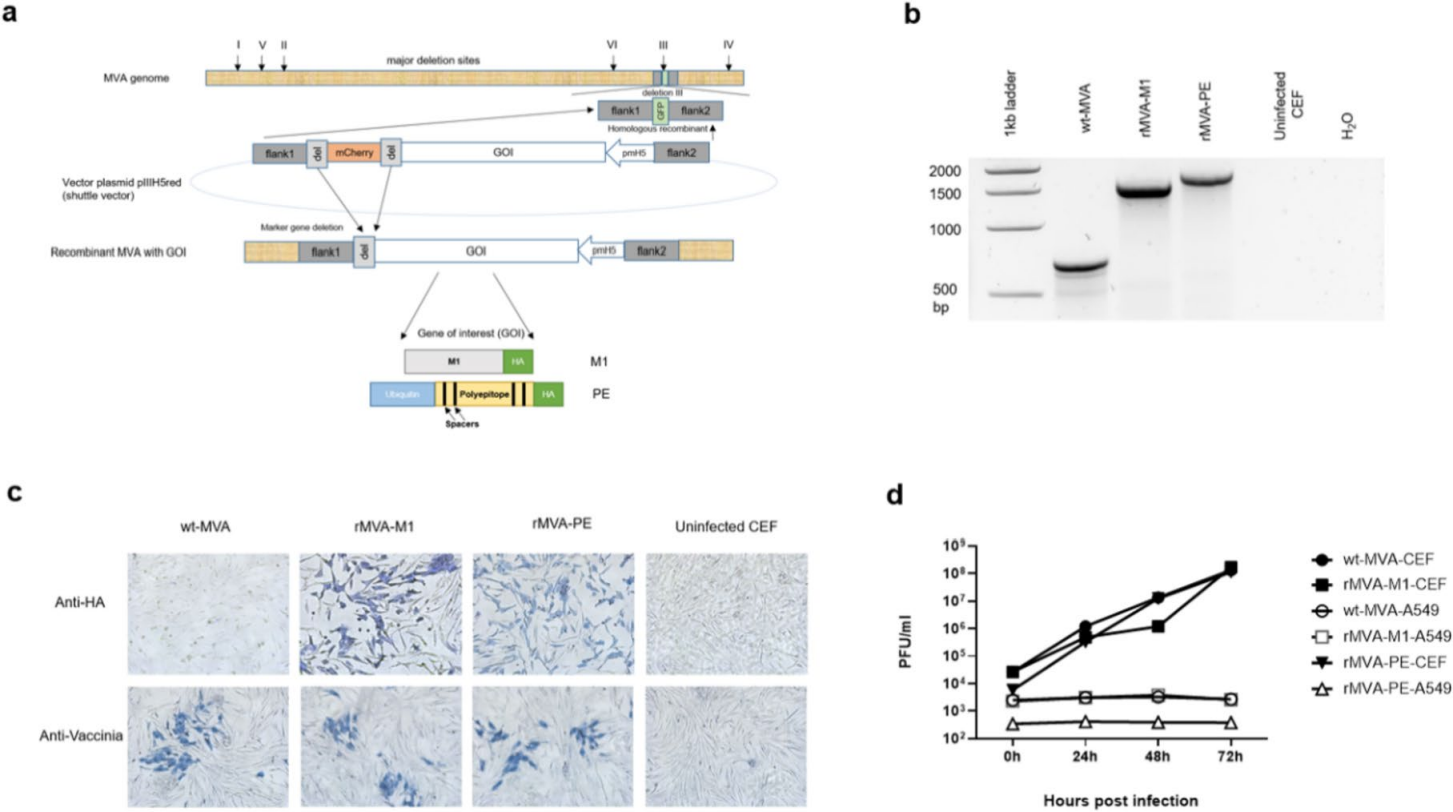
Construction and genetic characterisation of rMVAs. (a) Schematic diagram of the MVA genome with the major deletion sites I to VI. The site of deletion III served for insertion of the gene of interest (GOI) sequence. GOI was controlled by the virus-specific promoter PmH5 and inserted via homologous recombination between MVA DNA sequences (flank-1 and flank-2) adjacent to deletion site III in the MVA genome and copies cloned in the MVA vector plasmid pIIIH5redK1L. Expression of the red fluorescent marker protein mCherry was used during plaque purification. Repetition of short flank-1 derived DNA sequences (del) served to remove the marker gene by intragenomic homologous recombination (marker gene deletion). Construction of the artificial poly-epitope gene (PE) is shown in yellow. The epitopes of interest were fused to ubiquitin (blue) and separated by spacer sequences (black). The addition of an HA-tag (green) serves for easy monitoring of gene expression. For control purposes also the M1 gene from IAV A/Porto Rico/8/34 was cloned similar to the artificial poly-epitope immunogen. (b) Proper insertion of the genes of interest into MVA and intragenomic deletion of the marker gene mCherry was confirmed by PCR analysis using viral DNA as template and deletion III site-specific oligonucleotide primers. PCR amplified a characteristic 1.5 kb DNA product from rMVA with M1 (rMVA-M1) genomic DNA and 1.7 kb polyepitope with rMVA-PE genomic DNA. The expected 0.762 kb DNA fragment was obtained from wt-MVA DNA, which used as a positive control for PCR. Uninfected CEFs and H_2_O were used as negative controls. A full picture of the agarose gel shown in the supplementary document. (c) Immunostaining to confirm protein expression. CEFs were infected with the respective rMVAs or wt-MVA or left untreated. Cells were subsequently stained with antibody preparations against the HA-tag (Anti-HA) or VACV protein A27L (Anti-Vaccinia) as indicated. (d) Multiple-step replication analysis of recombinant MVAs (rMVA-PE and rMVA-M1) and wt-MVA in CEFs and A549 cells. After infection at a moi of 1, cell cultures were collected at the indicated time points and infectivity was determined by titration on CEFs. rMVAs and wt-MVA replicated efficiently in CEF cells but failed to replicate in A549 cells.

The artificial genes encoding the poly-epitope immunogen fused to ubiquitin with spacer sequences or the M1 protein of IAV A/Porto Rico/8/34 (sequence shown in the supplementary document) were cloned into deletion site III of the MVA vector under the transcriptional control of the enhanced synthetic VACV early/late promoter PmH5 (Fig. 2a). PCR analysis of viral DNA confirmed proper insertion of the artificial poly-epitope and M1 genes (Fig. 2b) and the absence of wt-MVA after plaque purification. Amplicons were detected of sizes corresponding to the respective inserts: 1.5 kb for rMVA-M1 and 1.7 kb for the artificial poly-epitope gene with spacers (rMVA-PE). The expected 0.762 kb DNA fragment was obtained with wt-MVA DNA, which was used as a control. Uninfected CEFs and H_2_O were used as negative controls. The identity of the inserts was further confirmed by nucleotide sequencing.

To test protein expression of rMVAs, immunostaining was conducted (Fig. 2c). CEF cells grown in 24-well tissue culture plates were infected with rMVAs at a moi of 0.5. wt-MVA infected cells were used as a control. After 48 h incubation, cells were stained with anti-VACV antibody and anti-HA antibody to assess viral infection and HA expression. Cells infected with rMVA-PE showed clear staining with the anti-HA antibody, confirming expression of HA-tagged protein (Fig. 2c, upper panel). In contrast, no staining was observed when using the anti-HA antibody in wt-MVA-infected cells. Staining with anti-VACV antibody was observed in both rMVA-PE and wt-MVA-infected cells, indicating that both recombinant and wild-type viruses were able to infect the cells (Fig. 2c, lower panel).

To demonstrate that the gene insertions did not alter the replication deficiency of the recombinant MVA in human cells, multiple-step growth kinetics of the respective rMVAs and wt-MVA were determined in human A549 cells. Because MVA has a highly attenuated phenotype in mammalian cells, but still drives the expression of genes of interest, rMVA-PE, rMVA-M1 and wt-MVA did not replicate in A549 cells as expected (Fig. 2d). In contrast, the respective rMVAs displayed similar replication kinetics as wt-MVA in avian CEFs, indicating that insertion of the artificial poly-epitope gene or the M1 gene did not affect the replication capacity of rMVAs in CEFs, which is of importance for large scale production of the MVA-PE in avian cells (Fig. 2d).

### In vitro antigenicity of MVA-PE

**Fig. 3:**
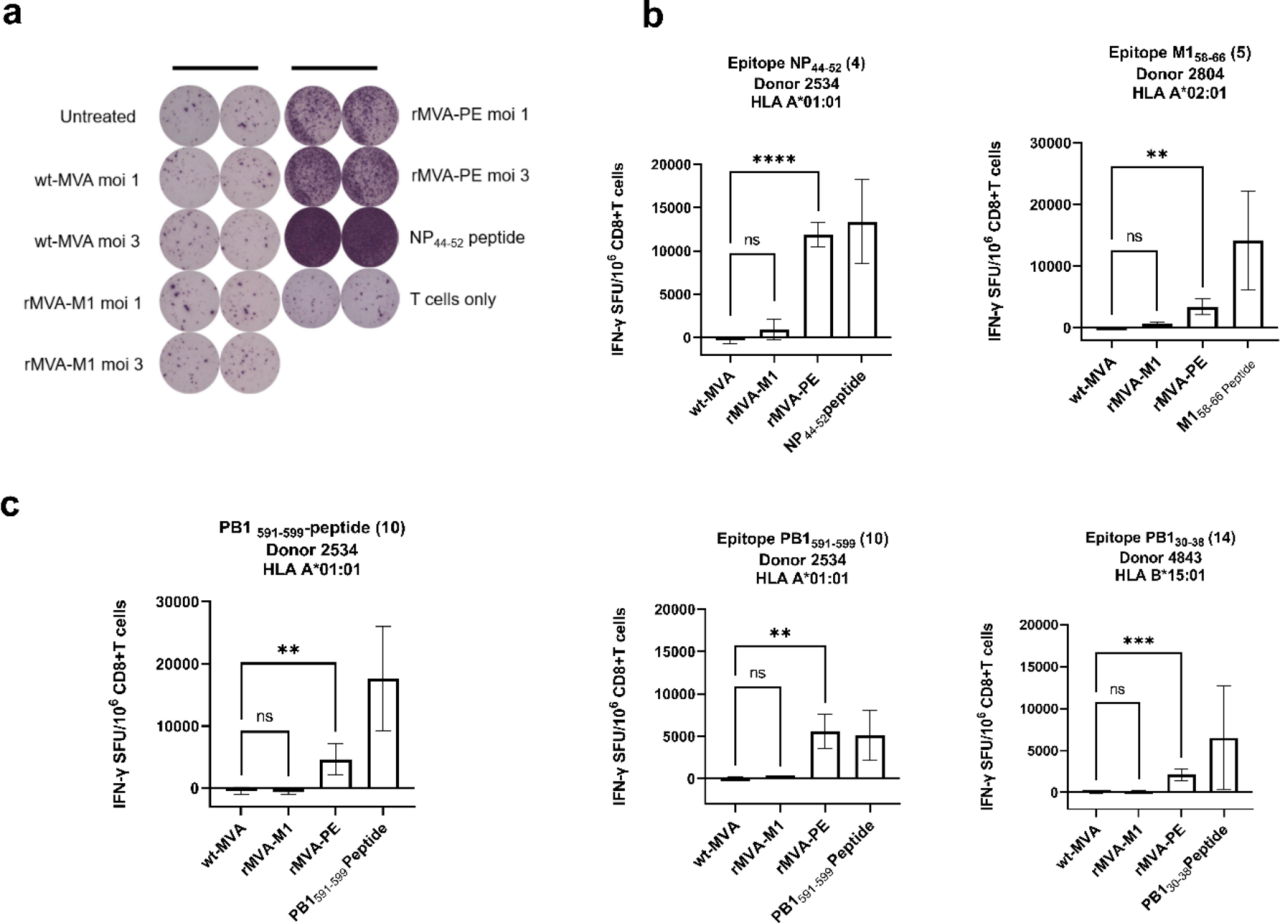
In vitro antigenicity test with rMVA constructs. (a) NP_44−52_ (CTELKLSDY)-specific CD8 + T cells incubated with rMVA- or wt-MVA-infected HLA-A*01 transgenic A549 cells at moi of 1 and 3. The CD8 + T cells response were measured by IFN-γ ELISpot assay. Only stimulation with rMVA-PE activated specific T cells. NP_44−52_ peptide loaded cells were used as a positive control. wt-MVA infected and uninfected transgenic A549-A*01 cells were used as negative controls, as well as T cells only. (b) Graphical representation of ELISpot data produced with CD8 + T cells directed against the four different peptides, PB1_30−38_ (YSHGTGTGY), PB1_591−599_ (VSDGGPNLY), M1_58−66_ (GILGFVFTL) and NP_44−52_ (CTELKLSDY) that were incubated with HLA-matched transgenic A549 cells infected (moi 3) with wt-MVA or rMVAs or loaded with peptide. The number behind the peptide represents the position of the peptide in a poly-epitope construct. Data are expressed as spot-forming unit (SFU) per 10^6^ CD8 + T cells after subtraction of values obtained with uninfected HLA-A*01 transgenic A549 cells. Data were obtained from two independent experiments performed in duplicate (*n* = 4). (c) Response of PB1_591−599_ (VSDGGPNLY)-specific CD8 + T cells after stimulation with autologous monocytes infected with rMVAs or wt-MVA infected (moi 3) or loaded with peptide measured by IFN-γ ELISpot assay. Activation of PB1_591−599_ -specific T cells was observed with rMVA-PE but not with wt-MVA or MVA-M1. Number behind the peptide represents the position of the peptide in a poly-epitope construct. Data are expressed as spot-forming unit (SFU) per 10^6^ CD8 + T cells after subtraction of values obtained with uninfected monocytes. Data derived from two independent experiments performed in duplicate (*n* = 4). Means ± standard deviation (SD) are shown. ∗∗, *p* < 0.01; ∗∗∗, *p* < 0.001; ∗∗∗∗, *p* < 0.0001; ns, not significant. p values of all shown significance indicators were determined by Two-way ANOVA and Tukey’s multiple comparison test.

To assess if the artificial poly-epitope fusion protein is processed and the epitopes liberated from the proteins and presented to virus specific T cells, HLA-transgenic A549 cells were infected with rMVA-PE, wt-MVA, left untreated or were loaded with peptide and used for stimulation of CD8 + T cells that were expanded in a peptide or virus-specific way. Activation of the T cells was determined by IFN-γ ELISpot and a representative result is shown for T cells directed to the HLA-A*01:01 restricted NP_44−52_ peptide in Fig. 3a. Stimulation with HLA-A*01:01-transgenic A549 cells infected with wt-MVA or rMVA-M1, which do not contain the epitope, did not activate NP_44−52_-specific T cells. In contrast, NP_44−52_ peptide-loaded cells and cells infected rMVA-PE activated NP_44−52_-specific T cells efficiently. This experiment was performed with PBMCs obtained from three different donors who were positive for HLA-A*01:01, HLA-A*02:01 or HLA-B*15:01, stimulated with four different peptides, NP_44−52_, PB1_591−599_ (both HLA-A*01:01), M1_58−66_ (HLA-A*02:01) and PB1_30−38_ (HLA-B*15:01), respectively. The results of the four experiments are shown in Fig. 3b. Each time, a response was achieved with rMVA-PE-infected A549 cells expressing the corresponding HLA-allele and after peptide loading, but not after stimulation with wt-MVA-infected A549 cells. M1_58−66_-specific T cells were also activated after stimulation with HLA-A*02:01 transgenic A549 cells infected with rMVA-M1, which harbours the corresponding epitope, although the response with peptide loaded cells was stronger. Of special interest, after stimulation with rMVA-PE infected HLA-matched A549 cells, a response was observed with T cells specific to all four peptides tested, NP_44−52_, M1_58−66_, PB1_591−599_ and PB1_30−38_, which are located on position 4th, 5th, 10th and 14th in the poly-epitope string (Fig. 3b). This indicates that the liberation of the respective epitopes from the artificial immunogen is independent of their position in the poly-epitope sequence. To confirm that also in professional antigen presenting cells (APCs) processing of the poly-epitope sequence takes place, we also infected autologous monocytes of one donor with the rMVA-PE and observed again a response with T cells directed to the PB1_591−599_ epitope after stimulation with monocytes infected with MVA-PE, but not with wt-MVA-infected monocytes (Fig. 3c).

**Fig. 4:**
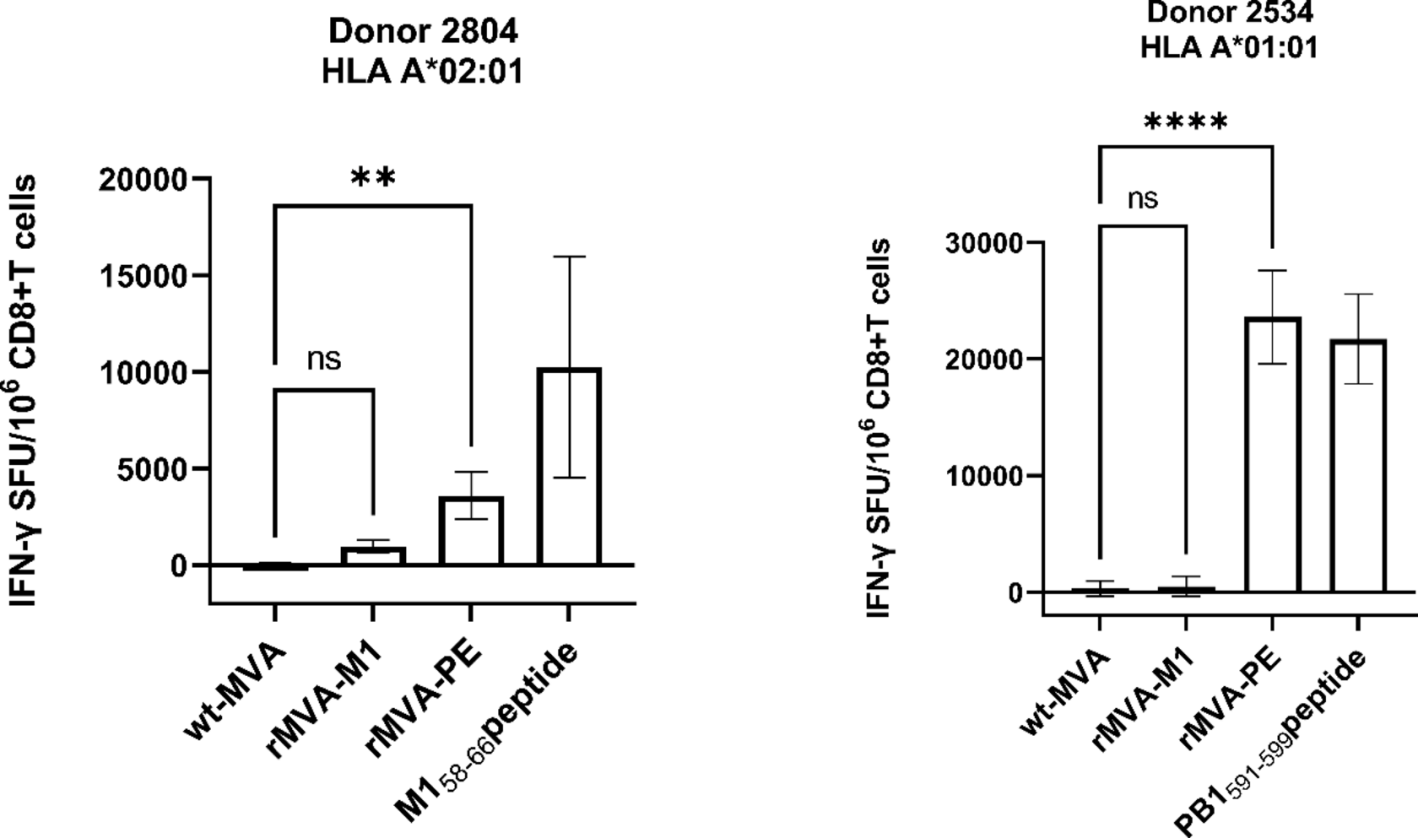
In vitro antigenicity test with rMVA constructs using CD8 + T cells expanded after stimulation with IAV. IAV-specific CD8 + T cells obtained from two blood donors (HLA-A*02:01 and A*01:01, respectively) were incubated with corresponding HLA- transgenic A549 cells infected with the indicated rMVAs or wt-MVA and their response was measured by IFN-γ ELISpot assay. M1_58−66_ and PB1_591−599_ loaded A549 cells were included as positive controls. Data are expressed as spot-forming unit (SFU) per 10^6^ CD8 + T cells after subtraction of values obtained with uninfected A549 cells. Data derived from two independent experiments performed in duplicate (*n* = 4). Means ± SD are shown. ∗∗, *p* < 0.01; ∗∗∗∗, *p* < 0.0001; ns, not significant. p values were determined by Two-way ANOVA and Tukey’s multiple comparison test.

We also used T cells obtained after stimulation of PBMCs of two blood donors with IAV Ned98 (H3N2). These virus-specific T cells responded to restimulation with HLA-matched A549 cells infected with rMVA-PE or loaded with peptide (Fig. 4). Also, some reactivity was observed for rMVA-M1 with the donor who was HLA-A*02:01 positive and was able to mount a response to the immunodominant M1_58−66_ epitope located within the M1 protein.

### In vitro immunogenicity of rMVA-PE

**Fig. 5:**
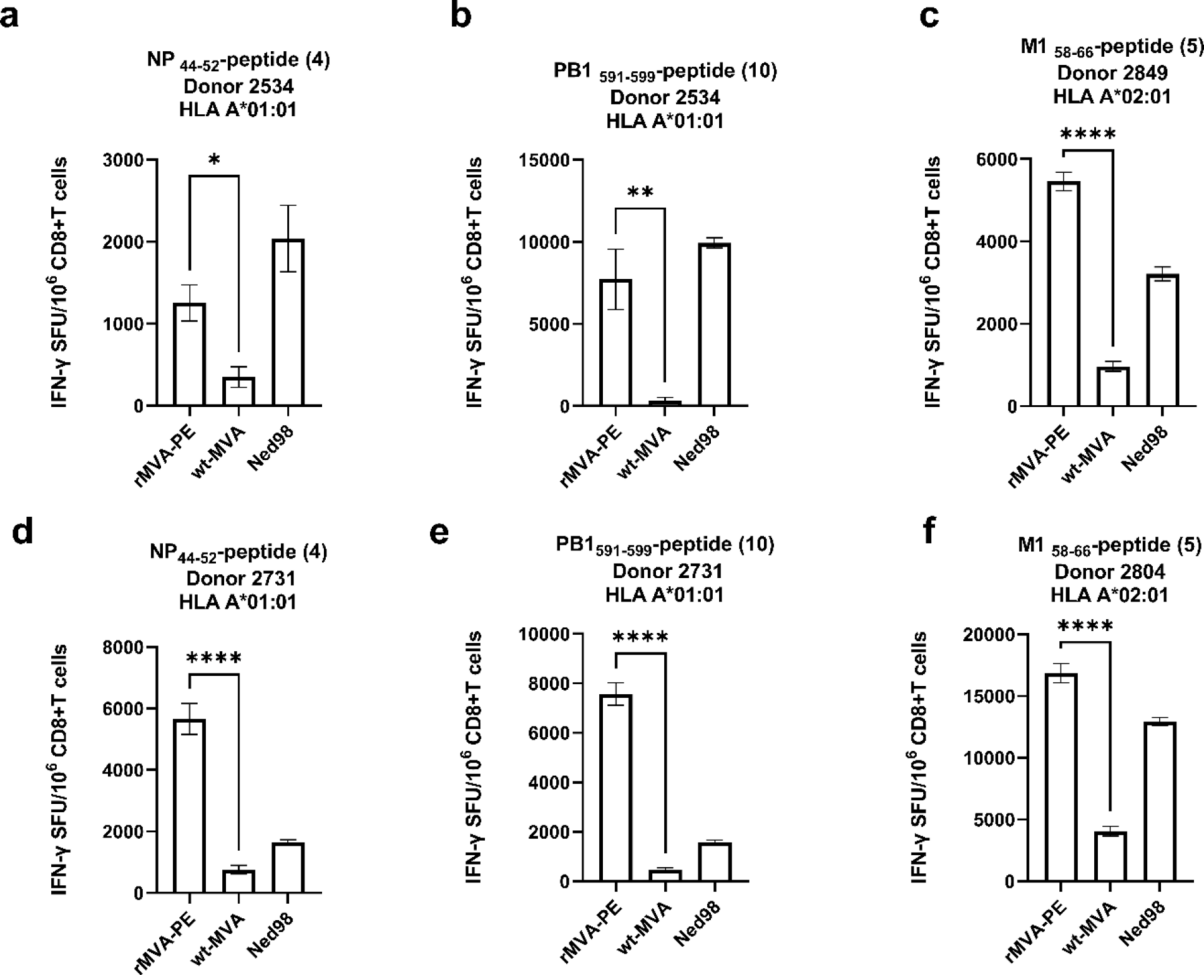
In vitro immunogenicity of rMVA-PE. In vitro immunogenicity of rMVA-PE was assessed by stimulating PBMCs of HLA-A*01:01 and A*02:01 positive blood donors with rMVA-PE, wt-MVA or IAV Ned98 (moi of 1) as indicated. After 12 days, the frequency of peptide-specific CD8 + T cells was determined by IFN-γ ELISpot assay after re-stimulation with corresponding HLA- transgenic A549 cell loaded with HLA-A*01:01 peptides NP_44−52_ (CTELKLSDY) (a, d) and PB1_591−599_ (VSDGGPNLY) (b, e) or HLA-A*02:01 peptide M1_58−66_ (GILGFVFTL) (c, f). The number behind the peptide represents the position of the peptide in a poly-epitope construct. Means ± SD are shown (*n* = 3). ∗, *p* < 0.05; ∗∗, *p* < 0.01; ∗∗∗∗, *p* < 0.0001. p values were determined by Two-way ANOVA and Tukey’s multiple comparison test.

To test the in vitro immunogenicity of rMVA-PE, PBMCs obtained from two HLA-A*01:01 and two HLA-A*02:01 positive donors were stimulated with rMVA-PE and IAV Ned98 (H3N2) to activate IAV-specific memory T cells. Subsequently, the frequency of CD8 + T cells from these expansion cultures specific for the NP_44−52_, PB1_591−599_ (both HLA-A*01:01) and M1_58−66_ epitopes was determined by IFN-γ ELISpot assay. As shown in Fig. 5, in most cases the induction of peptide-specific T cells with rMVA-PE was as good or even better as with stimulation with IAV. rMVA-PE induced potent responses to the NP_44−52_ and PB1_591−599_ epitopes in PBMC of HLA-A*01:01 positive donors (Fig. 5a, d, b and e). The immunodominance hierarchies of the MVA-PE induced response to these two peptides tested were also similar to the response induced with IAV. In the HLA-A*02:01 positive donors, rMVA-PE induced a T cell response to the immunodominant M1_58−66_ epitope (Fig. 5c, f). Thus, with rMVA-PE, IAV-specific memory T cell responses could be induced in vitro, that resembled the responses induced with IAV.

### Immunogenicity of rMVA-PE in-vivo

**Fig. 6:**
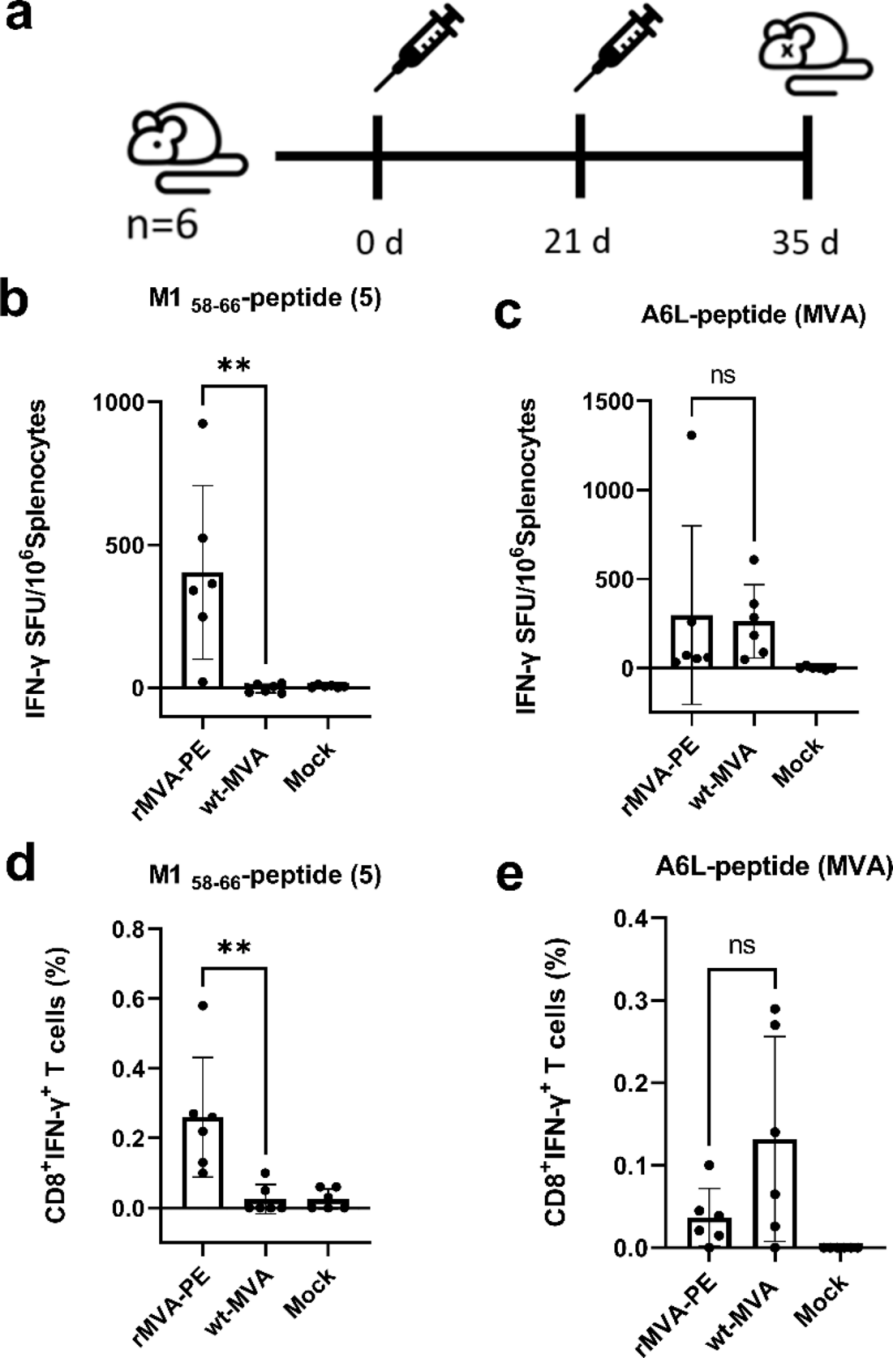
In vivo immunogenicity of rMVA-PE. (a) Experimental protocol. HLA-A2.1-/HLA-DR1-transgenic H-2 class I-/class II-knockout mice (*n* = 6) were immunized intramuscularly with 10^7^ PFU of wt-MVA, or rMVA-PE, or with vaccination buffer (Mock) on days 0 and 21 (A). On day 35, splenocytes were isolated and tested by ELISpot assay to detect wt-MVA and rMVA-PE-induced CD8 + T cell responses (b, c). Splenocytes were incubated with A*02:01 restricted influenza virus-specific peptide M1_58−66_ (b) which is in the 5th position in the poly-epitope vaccine or MVA-specific peptide A6L_6−14_ (c). Data are expressed as spot-forming unit (SFU) per 10^6^ splenocytes after subtraction of background control. Frequencies of IFN-γ producing CD8 + T cells were also determined by intracellular cytokine staining and flow cytometry (d, e). Splenocytes were stimulated with the HLA-A*02:01 restricted peptide M1_58−66_ or MVA-specific peptide A6L. The data points represent individual mice (*n* = 6). The graphs represent the mean ± SD. n.s. not significant (*p* > 0.05); **, *p* < 0.01 (Mann-Whitney test).

To assess the immunogenicity of rMVA-PE in vivo, humanized HLA-A2.1-/HLA-DR1-transgenic *H-2 class I-/class II*-knockout mice (HLA-A*02:01) were immunized twice intramuscularly (days 0 and 21) with 10^7^ PFU of wt-MVA, rMVA-PE or mock-immunized with vaccination buffer (Fig. 6a). Two weeks after the booster vaccination, splenocytes were tested for the presence of IAV- and MVA- specific CD8 + T cell responses by IFN-γ ELISpot assay. Vaccination with rMVA-PE induced T cell responses directed to the HLA-A*02:01 restricted IAV M1_58−66_ epitope that was not observed after vaccination with wt-MVA or in mock-vaccinated mice (Fig. 6b). As expected, both with wt-MVA and rMVA-PE, T cell responses to the MVA-derived A6L_6−14_ peptide were observed (Fig. 6c). Further characterization of rMVA-PE induced T cell responses by flow cytometry showed that IFN-γ^+^ cells responding to restimulation with the M1_58−66_ peptide or the A6(L)_6−14_ peptide were indeed CD8 + T cells (Fig. 6d-e). Thus, rMVA-PE is highly immunogenic in HLA-A2.1-/HLA-DR1-transgenic H-2 class I-/class II-knockout mice and induces influenza virus-specific CD8 + T cell responses.

## Discussion

In the present study, we designed a synthetic immunogen containing 20 conserved CD8 + T cell IAV epitopes, fused the artificial sequence to ubiquitin and added a AAY spacer sequence between the potential epitopes (rMVA-PE). Using MVA for expression of the poly-epitope immunogen, liberation of individual epitopes and presentation to virus-specific T cells was demonstrated. Immunogenicity of rMVA-PE was confirmed in vitro after stimulation of PBMC obtained from HLA-matched study subjects. Subsequent evaluation of rMVA-PE in vivo by using humanized HLA-A2.1-/HLA-DR1-transgenic H-2 class I-/class II-knockout mice (HLA-A*02:01) confirmed the observed in vitro results. It was concluded that delivery of a poly-epitope immunogen comprising of selected conserved T cell epitopes is an attractive vaccination strategy for the induction of cross-reactive IAV-specific CD8 + T cell responses.

For the induction of broadly cross-reactive IAV-specific CD8 + T cell responses, it is imperative that the epitopes are highly conserved and shared by a variety of influenza viruses of various subtypes and originating from various animal species. We considered all epitopes collected in the IEDB of human influenza viruses, but also avian and swine influenza viruses because birds and pigs are known as important reservoirs of influenza A viruses and may act as “mixing vessels” for genetically reassorted viruses, respectively. Because the vast majority of CTL epitopes are 8-11mer^54,55^, our search focused on epitopes of this length located in the most important targets for influenza virus-specific CD8 + T cell responses, the structural internal proteins NP and M1, and the polymerases PB1, PB2, and PA^22,23,25,56^. Finally, HLA polymorphism in the human population was considered, which complicates the design of vaccines based on peptide sequences that aim at the induction of virus-specific T cell responses. The 20 epitopes that were ultimately selected for introduction into the artificial immunogen, displayed an HLA coverage of 99.5%, indicating that the poly-epitope-based vaccine would be immunogenic in the vast majority of human subjects worldwide.

For the delivery of the artificial poly-epitope immunogens we choose the replication-deficient pox viral vector MVA, because of its known safety profile confirmed in various clinical trials and for its capacity to induce humoral and cellular immune responses to the expressed protein of interest^57,58^. We confirmed that insertion of the poly-epitope sequence into MVA did not alter its replication deficiency in mammalian cells. For optimal docking to the proteasome and subsequent antigen processing, we fused the poly-epitope string to ubiquitin^37–40^. The C-terminus of ubiquitin was modified by substituting glycine with valine at position 76 to prevent the cytosolic cleavage of ubiquitin, ensuring that the fusion protein is effectively targeted for proteasomal degradation^37,39,59^. Of interest, multiple attempts to rescue recombinant MVA expressing the poly-epitope string without fusion to ubiquitin failed, whereas rMVA expressing the ubiquitin-PE fusion products were readily generated. Apparently, fusion to ubiquitin was required to prevent over expression of potentially toxic products intracellularly. Research on poly-epitope vaccines has shown that spacer sequences between epitopes are crucial for enhancing immunogenicity and efficacy. Bazhan et al. demonstrated that including optimized spacers for proteasomal processing of a poly-epitope construct resulted in the most immunogenic vaccine^60^. Others found that adding spacers between epitopes significantly improved immunity to tumors and, when combined with ubiquitin targeting, led to the complete eradication of established tumors^61^. Schubert & Kohlbacher developed a framework for optimizing spacer length and sequence, resulting in a five-fold increase in predicted epitope recovery and a 44% reduction in neo-epitope generation^41^. These studies collectively emphasize the importance of spacer sequences in poly-epitope vaccine design and efficacy, which we applied in the present study.

Stimulation with HLA-transgenic A549 cells infected with rMVA-PE resulted in the activation of CD8 + T cells directed to four different epitopes, thus, demonstrating that the respective epitopes were liberated from the artificial immunogen, independent of their location in the poly-epitope string. Since upon administration in vivo, MVA targets professional antigen presenting cells^48^, we also demonstrated that rMVA-PE-infected autologous monocytes were able to present the PB1_591−599_HLA-A*01:01 restricted epitope to specific T cells. This activation is independent of the location of the epitope sequence in the poly-epitope construct^33,41^. To test whether rMVA-PE could induce recall in vitro T cell responses, PBMCs of four study subjects were stimulated with rMVA-PE or influenza virus and the expanded T cells tested for recognition of three different peptides. rMVA-PE induced peptide-specific T cell responses similar to those induced with influenza virus. In some study subjects, even the immunodominance hierarchies of the responses against the respective peptides were similar. In these experiments, pre-existing memory CD8 + T cells induced by prior IAV infections were reactivated and expanded after infection of the PBMC with rMVA-PE. Although not tested, most likely the rMVA-PE-induced T cells also display cytoxic activity, since the threshold for cytokine induction is equal or higher than that for the induction of cytotoxic activity, which is based on degranulation and granzyme B/perforin release^62,63^.

The in vivo immunogenicity of rMVA-PE was tested in HLA-A2.1-/HLA-DR1-transgenic mice^50,64^. Both rMVA-PE and wt-MVA vector control induced a CD8 + T cell response to the MVA-derived peptide A6L_6−14_. In contrast, only with rMVA-PE a CD8 + T cell response was observed against the influenza virus HLA-A*02:01 restricted M1_58−66_epitope and an IAV-specific T cell response (data not shown). Of note, responses to the other HLA-A*02:01 restricted epitopes were not detectable (data not shown), which might be explained by their subdominant nature^65,66^. The immunogenicity outcomes in HLA-A*02:01 transgenic mice may not precisely mirror those of HLA-A*02:01 positive humans, due to the evolutionary absence of HLA in mice and the resultant disparities in T cell selection and TCR repertoire between mice and humans. Consequently, the magnitude and quality of the specific CD8 + T cells response may be underestimated in (naïve) HLA-A*02:01 transgenic mice compared to those in humans. Furthermore, the human population at large has been primed by previous infections^67^ and therefore, immunization with rMVA-PE will lead to recall responses, in contrast to its use in immunologically naïve mice.

Previously it has been shown that a single vaccination with recombinant MVA expressing the influenza A virus NP and M1 genes (MVA-NP + M1) boosted T cell responses and reduced symptoms and duration of viral shedding upon experimental infection of humans study subjects^68^. In addition, MVA-NP + M1 was tested in two clinical trials in combination with quadrivalent influenza vaccine (QIV) in older adults. Although MVA-NP + M1-vaccination induced significant T-cell responses against conserved influenza antigens, it did not improve protection against influenza when combined with QIV in adults aged 65 and older^69,70^. Possibly, QIV-induced antibody responses may have obscured the effect of the rMVA induced T cell responses. Alternatively, MVA-NP + M1 may have induced a T cell response only in a proportion of the study subjects due to HLA restriction. Our vaccine candidate is fundamentally different from MVA-NP + M1. The use of an artificial poly-epitope immunogen as we have designed and evaluated in the present study, offers several advantages. A wider array of highly conserved epitopes was included, which may afford broader cross-reactivity and more robust protection against multiple IAV subtypes. Furthermore, the poly-epitope approach may address a possible limitation of some other vaccine candidates, which is variability in T cell responses bewteen subjects of different HLA haplotypes^71^. By incorporating epitopes with broad HLA coverage, our vaccine candidate may induce more consistent CD8 + T cell responses across diverse populations, enhancing its potential as a more universal influenza vaccine. Further investigations have to confirm the universal applicability of the MVA-PE vaccine across all HLA haplotypes and influenza A viruses of various subtypes and origins, which is a limitation of the present study.

In conclusion, we designed an artificial immunogen aiming at the induction of cross-reactive IAV-specific CD8 + T cell responses. In this study, we demonstrated proof of principle using MVA as vaccine delivery system, which is a safe and scalable vaccine production platform and used as vaccine against mpox^72^and tested as recombinant vector against other infectious diseases^73–75^. Alternatively, the synthetic poly-epitope antigen can also be delivered by other antigen delivery systems, such as mRNA-based vaccine preparations^76^. We anticipate that the use of rMVA-PE will induce cross-reactive CD8 + T cell responses to most IAVs, regardless of their subtype and species of origin, including influenza viruses of the H5N1 subtype detected in dairy cattle in the USA that also caused human cases, because 17 out of the 20 epitopes are identical in these viruses. It is expected that vaccine-induced cell-mediated immunity could reduce severity and duration of disease caused by infection with newly emerging IAVs or as adjunct to conventional influenza vaccines, and therefore, further clinical testing of the MVA-PE vaccine candidate is waranted.

## Electronic supplementary material

Below is the link to the electronic supplementary material.

Supplementary Material 1

## Data Availability

All data generated or analysed during this study are included in this published article (and its Supplementary Information files).
