# Supplementary material for: Design and evaluation of a poly-epitope based vaccine for the induction of influenza A virus cross-reactive CD8 + T cell responses
Source: Sci Rep. 2025 Mar 27;15:10586. doi: 10.1038/s41598-025-95479-9 (PMC11950192; doi:10.1038/s41598-025-95479-9)
Supplement: Supplementary file 1 — Supplementary Material 1 [file 41598_2025_95479_MOESM1_ESM.docx]

M1 from Influenza A virus (A/Puerto Rico/8/1934(H1N1) (PR8), Accession number: NP_040978

atg agt ctt cta acc gag gtc gaa acg tac gta ctc tct atc atc ccg tca ggc cct ctc aaa gcc gag atc gca cag aga ctt gaa gat gtc ttt gca ggg aag aac acc gat ctt gag gtt ctc atg gaa tgg cta aag aca aga cca atc ctg tca cct ctg act aag ggt att tta gga ttt gtg ttc acg ctc acc gtg ccc agt gag cga gga ctg cag cgt aga cgc ttt gtc caa aat gcc ctt aat ggg aac ggt gat cca aat aac atg gac aaa gca gtt aaa ctg tat agg aag ctc aag agg gag ata aca ttc cat ggt gcc aaa gaa atc tca ctc agt tat tct gct ggt gca ctt gcc agt tgt atg ggc ctc ata tac aac agg atg ggt gct gtg acc act gaa gtg gca ttt ggc ctg gta tgt gca acc tgt gaa cag att gct gac tcc cag cat cgg tct cat agg caa atg gtg aca aca acc aat cca cta atc aga cat gag aac aga atg gtt tta gcc agc act aca gct aag gct atg gag caa atg gct gga tcg agt gag caa gca gca gag gcc atg gag gtt gct agt cag gct aga caa atg gtg caa gcg atg aga acc att ggg act cat cct agc tcc agt gct ggt ctg aaa aat gat ctt ctt gaa aat ttg cag gcc tat cag aaa cga atg ggt gtg cag atg caa cgg ttc aag TACCCATACGAT GTTCCAGATTACGCTTAA

M1 Protein sequences:

MSLLTEVETYVLSIIPSGPLKAEIAQRLEDVFAGKNTDLEVLMEWLKTRPILSPLTKGILGFVFTLTVPSERGLQRRRFVQNALNGNGDPNNMDKAVKLYRKLKREITFHGAKEISLSYSAGALASCMGLIYNRMGAVTTEVAFGLVCATCEQIADSQHRSHRQMVTTTNPLIRHENRMVLASTTAKAMEQMAGSSEQAAEAMEVASQARQMVQAMRTIGTHPSSSAGLKNDLLENLQAYQKRMGVQMQRFKYPYDVPDYA-

Poly-epitope (PE) (Spacer sequences are underlined):

atgCAGATCTTCGTGAAGACTCTGACTGGTAAGACCATCACCCTAGAGGTTGAGCCCAGTGACACCATCGAGAATGTCAAGGCAAAGATCCAAGATAAGGAAGGCATCCCTCCTGACCAGCAGAGGCTGATCTTTGCTGGAAAACAGCTGGAAGATGGGCGCACCCTGTCTGACTACAACATCCAGAAAGAGTCCACCCTGCACCTGGTGCTCCGTCTCAGAGGTGTA ata ttg aga ggg tcg gtt gct cac aag gcggcgtat ttc ctg ctg atg gat gcc tta aaa tta gcggcgtat agc agg tac tgg gcc ata agg acc aga gcggcgtat tgc acc gaa ctc aaa ctc agt gat tat gcggcgtat ggg att tta gga ttt gtg ttc acg ctc gcggcgtat tct atc atc ccg tca ggc cct ctc aaa gcggcgtat gcc agt tgt atg ggc ctc ata tac gcggcgtat ttc atg tat tca gat ttt cac ttc atc gcggcgtat ttt gtg cga caa tgc ttc aat ccg atg gcggcgtat gtc tcc gac gga ggc cca aat tta tac gcggcgtat ttc ctt aag gat gta atg gag tca atg gcggcgtat cgt ggg atc aat gat cgg aac ttc tgg gcggcgtat atg atg atg ggc atg ttc aat atg tta gcggcgtat tac agc cat ggg aca gga aca gga tac gcggcgtat cat tcc aat ttg aat gat gca act tat gcggcgtat agg agg tct gga gcc gca ggt gct gca gtc aaa gcggcgtat ctt cta acc gag gtc gaa acg tac gta gcggcgtat atg gtt tta gcc agc act aca gct aag gcggcgtat aat atg tta agc act gta tta ggc gtc gcggcgtat gaa ctg aga agc agg tac tgg gcc ata TACCCATACGATGTTCCAGATTACGCTTAA

Protein (each epitope shown in bold):

MQIFVKTLTGKTITLEVEPSDTIENVKAKIQDKEGIPPDQQRLIFAGKQLEDGRTLSDYNIQKESTLHLVLRLRGV**ILRGSVAHK**AAY**FLLMDALKL**AAY**SRYWAIRTR**AAY**CTELKLSDY**AAY**GILGFVFTL**AAY**SIIPSGPLK**AAY**ASCMGLIY**AAY**FMYSDFHFI**AAY**FVRQCFNPM**AAY**VSDGGPNLY**AAY**FLKDVMESM**AAY**RGINDRNFW**AAY**MMMGMFNML**AAY**YSHGTGTGY**AAY**HSNLNDATY**AAY**RRSGAAGAAVK**AAY**LLTEVETYV**AAY**MVLASTTAK**AAY**NMLSTVLGV**AAY**ELRSRYWAI**YPYDVPDYA-


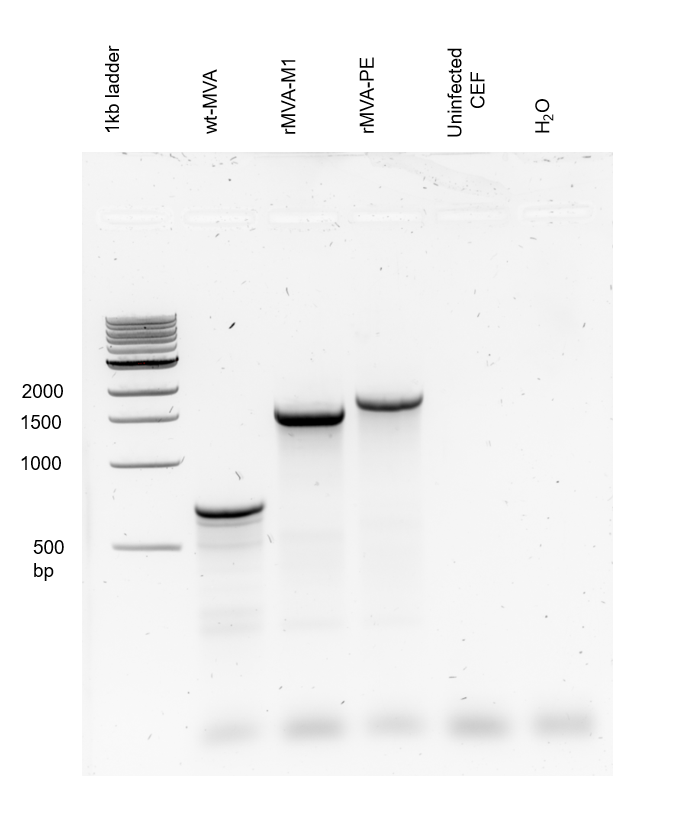


Full-length agarose gel (cropped gel is shown in Fig 1b)
